# Optical Coherence Tomography Angiography Reveals Distinct Retinal Structural and Microvascular Abnormalities in Cerebrovascular Disease

**DOI:** 10.3389/fnins.2020.588515

**Published:** 2020-10-02

**Authors:** Xiayin Zhang, Hui Xiao, Chunxin Liu, Sanxin Liu, Lanqin Zhao, Ruixin Wang, Jinghui Wang, Ting Wang, Yi Zhu, Chuan Chen, Xiaohang Wu, Duoru Lin, Wei Qiu, Patrick Yu-Wai-Man, Zhengqi Lu, Haotian Lin

**Affiliations:** ^1^State Key Laboratory of Ophthalmology, Zhongshan Ophthalmic Center, Sun Yat-sen University, Guangzhou, China; ^2^Department of Neurology, Psychological and Neurological Diseases Research Centre, The Third Affiliated Hospital, Sun Yat-sen University, Guangzhou, China; ^3^Department of Molecular and Cellular Pharmacology, University of Miami Miller School of Medicine, Miami, FL, United States; ^4^Sylvester Comprehensive Cancer Center, University of Miami Miller School of Medicine, Miami, FL, United States; ^5^Cambridge Centre for Brain Repair and MRC Mitochondrial Biology Unit, Department of Clinical Neurosciences, University of Cambridge, Cambridge, United Kingdom; ^6^Cambridge Eye Unit, Addenbrooke’s Hospital, Cambridge University Hospitals, Cambridge, United Kingdom; ^7^Moorfields Eye Hospital, London, United Kingdom; ^8^UCL Institute of Ophthalmology, University College London, London, United Kingdom; ^9^Center of Precision Medicine, Sun Yat-sen University, Guangzhou, China

**Keywords:** cerebrovascular disease, optical coherence tomography angiography, retinal vascular density, retinal structure, retinal microvasculature

## Abstract

Cerebrovascular disease (CeVD) is one of the leading global causes of death and severe disability. To date, retinal microangiopathy has become a reflection of cerebral microangiopathy, mirroring the vascular pathological modifications *in vivo*. To evaluate the retinal structure and microvasculature in patients with CeVD, we conducted a cross-sectional study in Zhongshan Ophthalmic Center and Department of Neurology of Third Affiliated Hospital, Sun Yat-sen University using optical coherence tomography angiography (OCTA). CeVD patients (*n* = 121; 238 eyes) and healthy controls (*n* = 44; 57 eyes) were included in the analysis. The CeVD group showed significant thinning of the peripapillary retinal nerve fiber layer (pRNFL) thickness in the temporal and nasal quadrants, and thinning of the macular ganglion cell-inner plexiform layer (GC-IPL) in the inferior quadrant, while macular microvasculature reduction was prominent in all nine quadrants. There were significant correlations between OCTA parameters, visual acuity, and transcranial doppler parameters in the CeVD group. The specific structural parameters combining microvasculature indices showed the best diagnostic accuracies (AUC = 0.918) to discriminate CeVD group from healthy controls. To conclude, we proved that OCTA reveals specific patterns of retinal structural changes and extensive macular microvascular changes in CeVD. Additionally, these retinal abnormalities could prove useful disease biomarkers in the management of individuals at high risk of debilitating complications from a cerebrovascular event.

## Introduction

Cerebrovascular disease (CeVD), affecting blood vessels that supply the brain, is one of the leading global causes of death and severe disability (Menken et al., 2000; Kochanek et al., 2019; Li et al., 2020). Despite extensive research on neuroimaging techniques, identifying patients at high risk of cerebrovascular complications is based on limited data that emphasize brain morphological changes (Paradise et al., 2018). In order to optimize treatment plans, a major research drive is the identification of potential biomarkers in other organs which reflect the underlying small vessel changes contributing to disease besides the brain (Arnould et al., 2018).

There is mounting evidence that the retina provides an accurate window into cerebrovascular and systemic vascular conditions (Stenkamp, 2015; Miesfeld and Brown, 2019; Rim et al., 2020). This is unsurprising given that the retina and the cerebrum share a common neurodevelopmental origin. Embryologically, besides the cerebrum, the forebrain neuroectoderm also contributes to the development of the retinal pigment epithelium and neural retina (Stenkamp, 2015; Miesfeld and Brown, 2019). Anatomically, both the anterior brain and the retina are supplied by the internal carotid artery (ICA; Rim et al., 2020). Although the brain vasculature has a complex intricate architecture, it has been suggested that retinal microangiopathy is a reflection of cerebral microangiopathy, mirroring the vascular pathological modifications *in vivo* (Goto et al., 1975).

The links between retinal parameters and CeVD have been extensively evaluated (Wong et al., 2001, 2002; Couper et al., 2002; Mitchell et al., 2005; McGeechan et al., 2009; Cheung et al., 2010; Hanff et al., 2014; Rajanala et al., 2020). Three major groups of retinal changes have been connected to CeVD, namely, features of hypertensive retinopathy, clinical retinal diseases, and retinal microvascular abnormalities (including arteriovenous nicking, focal arteriolar narrowing, and decreased arteriole-to-venule ratio; Wong et al., 2001; Mitchell et al., 2005; Cheung et al., 2010; Hanff et al., 2014). In addition, retinal microvascular abnormalities are correlated with an increased risk of stroke and stroke mortality (Wong et al., 2002). Longitudinal studies indicate that retinal vascular changes can predict patients at risk of progression to clinical CeVD (McGeechan et al., 2009; Rajanala et al., 2020). However, most studies were conducted using retinal photography (Wong et al., 2001; Couper et al., 2002; Mitchell et al., 2005; Cheung et al., 2010; Hanff et al., 2014), which cannot provide direct quantitative information on optic nerve structure and retinal vascular flow, and is less sensitive in the detection of retinal irregularities compared with optical coherence tomography (OCT; Goebel and Franke, 2006; Ouyang et al., 2013).

Optical coherence tomography angiography (OCTA) allows real-time quantitative evaluation of optic nerve structure and retinal vascular flow yielding greater image acquisition rates and sensitive measurements (Jia et al., 2015). Advanced OCTA techniques have become essential in the decision-making process of retinal disease management and can be used to identify CeVD patients (Kashani et al., 2017). Currently, the association between CeVD and abnormal findings in OCTA has not yet been investigated. In this study, we used OCTA to define whether specific patterns of damage involving the optic nerve and macular microvasculature is observed in patients with CeVD. The main outcome measures were peripapillary retinal nerve fiber layer (pRNFL) and macular ganglion cell-inner plexiform layer (GC-IPL) thickness, and macular vessel density (VD), and perfusion density (PD) in the superficial capillary plexus. We hypothesized that retinal structural and microvascular abnormalities in CeVD patients are quantifiable by OCTA, and these abnormalities could be considered as surrogate pathological markers for CeVD.

## Materials and Methods

### Subject Recruitment

Participants were volunteers who benefited from an OCTA examination from December 3, 2019 to May 3, 2020, and were recruited from Zhongshan Ophthalmic Center and Department of Neurology of The Third Affiliated Hospital, Sun Yat-sen University, Guangzhou, China. Written informed consent was obtained from all the participants. This study was approved by the Ethics Committee of Zhongshan Ophthalmic Center, Sun Yat-sen University (ethics board approval number: 2019KYPJ163), and it was conducted in accordance with the tenets of the Declaration of Helsinki.

Cerebrovascular disease patients were included if they were ≥18 years old. Two neurologists confirmed the diagnosis of CeVD, which in this study included intracranial hemorrhage and cerebral ischemia. Classification is based on the International Statistical Classification of Diseases and Related Health Problems 11th Revision from 2018 onward. Patients were excluded for the following reasons: (1) subjects diagnosed with other systemic diseases, including diabetes; (2) ocular diseases (including myopia (<-6 diopter), hyperopia (>6 diopter), the opacity of refractive media, age-related macular degeneration, glaucoma, hypertensive or diabetic retinopathy, optic disk pathology, and other eye pathology) or previous ocular surgery; (3) age < 18 years old; and (4) inability to provide informed consent. The criteria for inclusion in the healthy control group were no history of CeVD or other ocular and neurological diseases, with a normal fundus and visual acuity.

### Clinical Evaluation

All participants underwent an extensive ophthalmologic examination, including habitual visual acuity testing with a 6 m Snellen chart, slit-lamp biomicroscopy, fundus examination, fundus photography, and OCTA scans (Cirrus 5000, version 10.0; Zeiss Meditec, California, United States). All ophthalmological examinations were performed by a single well-trained clinician. For the visual acuity measurements, the refractive correction was used with the patient’s own spectacles under a chart luminance about 160 cd m^–2^. The standard termination rule is when a patient makes four or more mistakes on a line of five letters. Intraocular pressure measurement with Goldmann applanation tonometry, gonioscopy, and visual field testing by standard automated perimetry (SAP, Humphrey Field Analyzer; 30-2 Swedish interactive threshold algorithm; Carl Zeiss Meditec, Jena, Germany) were only considered to exclude suspected glaucoma or other retinopathy. The axial length measurement was only considered to exclude myopia (<-6 diopter) or microphthalmia.

All patients with CeVD were tested with computerized tomography scans and magnetic resonance imaging scans. The common carotid artery (CCA), ICA, and ophthalmic artery (OA) blood velocities were determined in some CeVD patients who agreed and were able to undergo bilateral 2 MHz transcranial doppler ultrasound (DWL, Doppler Box, Singen, Germany). The transcranial doppler ultrasound was performed by one experienced neurophysiology technician. Peak systolic velocity and mean velocity were recorded in cm/s.

### OCTA Acquisition and Processing

Optical coherence tomography angiography imaging was performed using a high-definition OCT and AngioPlex device (Cirrus 5000, version 10.0; Zeiss Meditec, California, United States) as described previously (Xiao et al., 2020a,b; Zhang et al., 2020). Briefly, an optic disk cube 200 × 200 scan mode was used for the pRNFL measurements, a macular cube with 512 × 128 scan mode was used to determine the macular thickness and GC-IPL thickness measurements, and angiography imaging was conducted centered at the macula with the 6 × 6 mm scan pattern (Figure 2A).

Peripapillary retinal nerve fiber layer thickness was measured using 3.46 mm diameter circles around the optic disk. The average pRNFL thickness and the thicknesses of the four-quadrant sectors (superior, temporal, inferior, and nasal) were analyzed. The GC-IPL thickness parameters evaluated were the average thickness within a 14.13 mm^2^ elliptical annular area region and the thicknesses in six quadrant sectors (superior, temporal-superior, temporal-inferior, inferior, nasal-inferior, and nasal-superior). Angiography scans were analyzed using Cirrus OCTA software (AngioPlex, version 10.0; Carl Zeiss Meditec). VD was calculated from the total length of perfused vasculature per unit area in a region of measurement, while PD was calculated from the total area of perfused vasculature per unit area in a region of measurement. The central foveal region was a region with a diameter of 1 mm, and the inner and outer rings had outer diameters of 3 and 6 mm, respectively. The VD and PD values of the nine quadrant sectors, the central, inner, and outer rings and the whole area were analyzed. The VD, PD and the foveal avascular zone (FAZ) of the superficial capillary plexus (SCP) were automatically measured by the in-built software from Carl Zeiss Meditec using optical microangiography algorithms. The software only calculates values for the SCP, which spans from the internal limiting membrane to the inner plexiform layer. The absence of motion artifacts was defined as no vessel doubling, vessel discontinuity/misalignment or lateral vessel displacement in the OCTA image. Images with a signal strength <7 and those with poor centration or segmentation errors were excluded from data analysis.

### Statistical Analyses

All statistical analyses were performed using software (SPSS, ver. 22.0; SPSS Inc., Chicago, IL, United States). The generalized estimating equations (GEE) method was used to adjust for age, gender, and the inter-eye correlation from the same participant. Pearson’s correlation was used to assess the associations between the OCTA parameters and the correlations between the OCTA values, visual acuity, and transcranial doppler values after testing using the GEE models. Logistic regression was employed to combine the diagnostic parameters into composite diagnostic indices. The area under the receiver operating characteristic curve (AUC) was used to calculate the diagnostic power of the diagnostic parameters. To compare the diagnostic capabilities of the parameters, the AUCs were compared using the method described by DeLong and colleagues (DeLong et al., 1988). A *P* value < 0.05 was considered statistically significant.

### Data Availability

The data that support the findings of this study is available from the corresponding author upon reasonable request.

## Results

### Demographic Data

**FIGURE 1:**
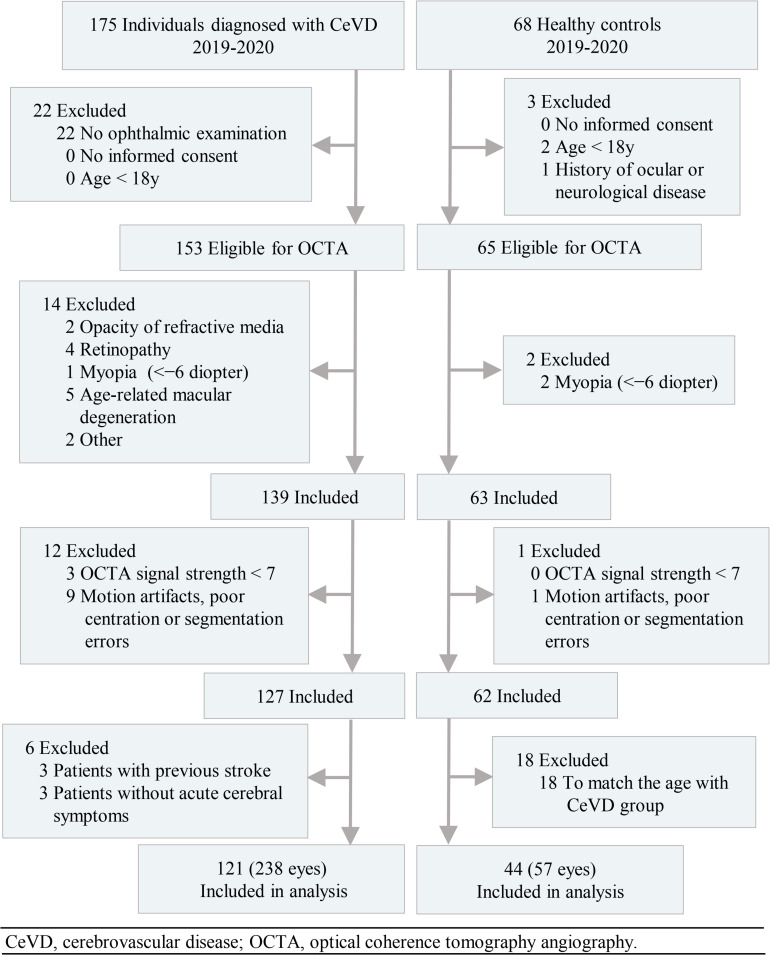
Flow diagram of the study population.

In total, the study enrolled 238 eyes of 121 subjects with CeVD and 57 eyes of 44 healthy controls in the final analysis (Figure 1). The age of the CeVD subjects was 56.00 ± 11.27y (mean ± SD), and the age of the healthy controls was 53.17 ± 7.29y (mean ± SD). Among the participants included in the final analysis, the causes of CeVD were intracranial hemorrhage (41 subjects; 79 eyes) and cerebral ischemia (80 subjects; 159 eyes). Table 1 shows the baseline characteristics of the study population.

**TABLE 1:** Demographic data and clinical characteristics.

Parameter	CeVD	Healthy controls	*P* value
Number of patients	121	44	–
Number of eyes	238	57	–
Age, mean (SD)	56.00 (11.27)	53.17 (7.29)	0.69
Sex, female:male*	93:28	25:19	<0.05
Habitual visual acuity, mean (SD) [Range]	0.55 (0.27) [0.29–1.30]	0.98 (0.12) [0.80–1.30]	<0.05

*Chi-square test, P value < 0.05 was considered to be statistically significant.CeVD, cerebrovascular disease; SD, standard deviation.

### Patterns of Loss in Retinal Structure and Microvasculature

**FIGURE 2:**
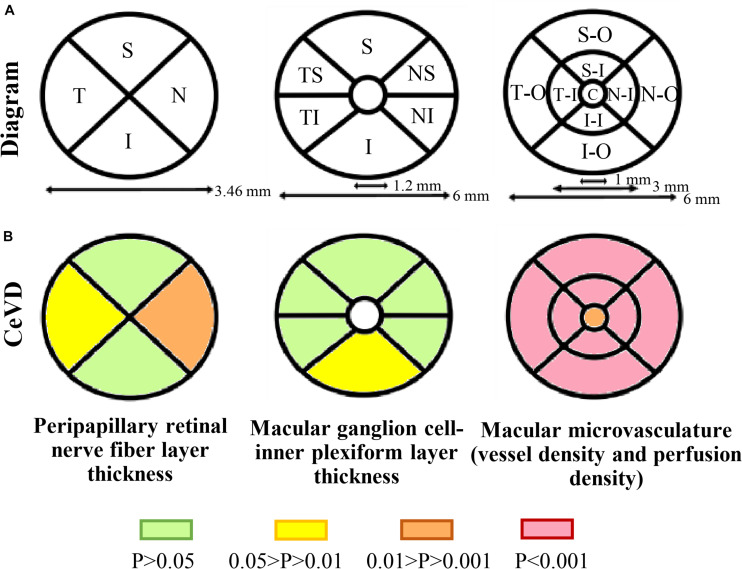
Retinal structural and microvasculature measurements. **(A)** Diagram of peripapillary and macular measurements using OCTA. Peripapillary retinal nerve fiber layer thickness was divided into four sectors, macular ganglion cell-inner plexiform layer thickness was divided into six sectors and macular microvasculature was divided into nine sectors. **(B)** Topographic damage in CeVD eyes. The thickness of the pRNFL was significantly reduced in the temporal and nasal quadrants, while the thickness of the GC-IPL was significantly reduced in the inferior quadrant. The CeVD group showed significantly reduced macular vessel density and perfusion density in all nine quadrants compared with the healthy controls. Macular microvasculature was measured in the superficial capillary plexus. Abbreviations: CeVD, cerebrovascular disease; S, superior; T, temporal; I, inferior; N, nasal; TS, temporal-superior; TI, temporal-inferior; NS, nasal-superior; NI, nasal-inferior; SO, superior-outer; TO, temporal-outer; IO, inferior-outer; NO, nasal-outer; SI, superior-inner; TI, temporal-inner; II, inferior-inner; NI, nasal-inner; and C, central.

The structural and microvascular parameters were evaluated using GEE models to adjust for age, sex, and within-subject inter-eye correlations (Table 2). The CeVD group showed significant thinning of the pRNFL at the temporal (*P* = 0.015) and nasal quadrants (*P* = 0.004). At the macula, the thickness of the GC-IPL was significantly reduced in the inferior quadrant in the CeVD group (*P* = 0.028). In addition, the CeVD group showed significantly reduced macular VD and PD compared with the healthy controls in all nine quadrants (Figure 2B). No significant differences were found in the whole macular thickness or the size of FAZ between patients with CeVD and healthy controls.

**TABLE 2:** Comparison of optical coherence tomography angiography parameters between patients with CeVD and healthy controls.

OCTA Parameters	CeVD versus healthy controls	
	**CeVD, mean (SD)**	**Healthy controls, mean (SD)**
Average pRNFL	96.51 (10.01)*	101.00 (7.78)
S pRNFL	121.26 (18.80)	124.46 (16.00)
T pRNFL	70.91 (12.50)*	77.54 (12.86)
I pRNFL	126.32 (20.35)	128.04 (18.55)
N pRNFL	67.60 (10.62)**	73.98 (15.78)
Whole macular thickness	248.62 (21.76)	244.90 (20.05)
Average GC-IPL	82.70 (7.78)	85.21 (5.92)
S GC-IPL	83.06 (9.18)	85.88 (6.36)
TS GC-IPL	81.92 (8.56)	83.04 (5.47)
TI GC-IPL	82.82 (8.23)	84.38 (5.79)
I GC-IPL	80.30 (8.25)*	83.65 (6.23)
NI GC-IPL	83.20 (7.81)	86.31 (6.19)
NS GC-IPL	85.17 (9.18)	87.92 (6.71)
Whole VD of superficial capillary plexus	16.37 (1.64)***	18.09 (0.94)
Central VD	6.93 (2.96)**	8.81 (2.84)
Inner VD	16.21 (2.11)***	18.19 (1.07)
Outer VD	16.79 (1.58)***	18.40 (0.92)
Whole PD of superficial capillary plexus	0.3979 (0.0435)***	0.4433 (0.0254)
Central VD	0.1536 (0.0693)**	0.1966 (0.0653)
Inner VD	0.3842 (0.0537)***	0.4320 (0.0284)
Outer VD	0.4113 (0.0425)***	0.4559 (0.0256)
FAZ area of superficial capillary plexus	0.3057 (0.1199)	0.2868 (0.1131)
Signal quality	8.89 (0.99)	9.16 (0.82)

The comparison of OCTA parameters between patients with CeVD and healthy controls was adjusted for age, sex, and within-subject inter-eye correlations. OCTA values are presented as the mean [standard deviation (SD)]. pRNFL, GC-IPL, and whole macular thicknesses are expressed in μm; VD and PD in mm^–1^; and FAZ area in mm^2^. *p < 0.05; **p < 0.01; and ***p < 0.001.CeVD, cerebrovascular disease; OCTA, optical coherence tomography angiography; SD, standard deviation; pRNFL, peripapillary retinal nerve fiber layer; GC-IPL, ganglion cell-inner plexiform layer; VD, vessel density; PD, perfusion density; FAZ, foveal avascular zone; S, superior; T, temporal; I, inferior; N, nasal; TI, temporal-inferior; TS, temporal-superior; NI, nasal-inferior; and NS, nasal-superior.

### Correlation Analysis Between OCTA, Visual Function and Transcranial Doppler Parameters

Since VD was strongly correlated with PD (*r* = 0.991, *P* < 0.0001), VD was used as a representative microvasculature manifestation in the following analysis. In CeVD, visual acuity was significantly correlated with the average pRNFL thickness (*r* = 0.194, *P* = 0.039). There was also a significant correlation between visual acuity and the superior-outer quadrant of the VD (*r* = 0.276, *P* = 0.018). No statistically significant correlations were found between visual acuity and the thickness of GC-IPL or the size of the FAZ area.

In addition, in 41 patients (33.9%) who finished the transcranial doppler ultrasound examinations, the peak velocity of the CCA was significantly correlated with the central VD (*r* = 0.423, *P* = 0.028, respectively). The peak velocity of the OA was significantly correlated with superior quadrant of the pRNFL (*r* = 0.351, *P* = 0.031). In addition, a significant correlation was found between visual acuity and the peak velocity of the OA (*r* = 0.412, *P* = 0.011).

### Diagnostic Accuracy of OCTA Parameters

The diagnostic capability of the OCTA parameters was calculated by the AUCs. GEE models were used to adjust for age, sex, and within-subject inter-eye correlations.

**FIGURE 3:**
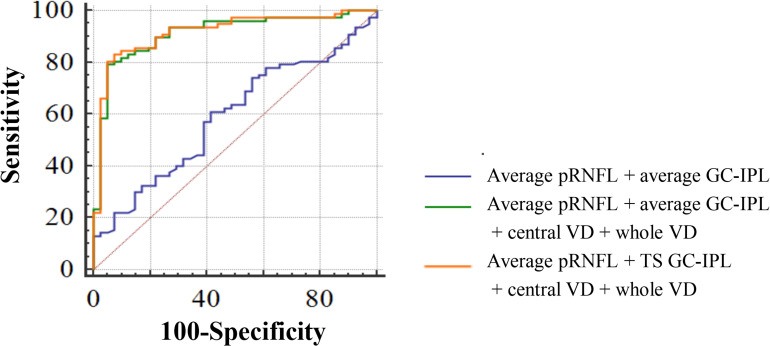
Diagnostic accuracy of optical coherence tomography angiography parameters in discriminating patients with cerebrovascular disease. AUC for average pRNFL + average GC-IPL = 0.590; AUC for average pRNFL + average GC-IPL + central VD + whole VD = 0.913; and AUC for average pRNFL + TS GC-IPL + central VD + whole VD = 0.918. Abbreviations: AUC, area under the curve; pRNFL, peripapillary retinal nerve fiber layer; GC-IPL, ganglion cell-inner plexiform layer; VD, vessel density; and TS, temporal-superior.

To discriminate CeVD from healthy controls, the structural OCT parameters (pRNFL average thickness and GC-IPL average thickness) combined with the macular angiography parameters (central VD and whole VD) significantly improved the diagnostic accuracy compared to only the structural parameters (*P* < 0.0001). After selecting the specific quadrants of pRNFL and GC-IPL with the best performance to replace the average thicknesses, the structural OCT parameters (pRNFL average thickness and temporal-superior quadrant thickness of GC-IPL) combined with the macular angiography parameters were the best discriminators between the CeVD and healthy control groups, with a diagnostic accuracy of 0.918 (Figure 3 and Table 3).

**TABLE 3:** Diagnostic ability of optical coherence tomography angiography parameters in distinguishing between patients with CeVD and healthy controls.

Diagnostic parameters	Average pRNFL + average GC-IPL	Average pRNFL + average GC-IPL + central VD + whole VD	Average pRNFL + TS GC-IPL + central VD + whole VD
AUC (95% CI)	0.59 (0.485∼0.695)	0.913 (0.857∼0.969)	0.918 (0.863∼0.972)
*P* values	–	<0.0001	0.5019
Sensitivity Specificity Correct indices	0.610 0.586 0.196	0.792 0.951 0.743	0.831 0.927 0.758

*P values were evaluated with the AUC on the left. The diagnostic parameters to differentiate patients with CeVD from healthy controls were derived from the logistic regression.CeVD: cerebrovascular disease; AUC: area under the curve; CI: confidence interval; pRNFL: peripapillary nerve fiber layer; GC-IPL: ganglion cell-inner plexiform layer; TS: temporal-superior; and VD: vessel density.*

## Discussion

Exploration of retinal vessel abnormalities may facilitate early detection for cerebrovascular events, and can potentially become the main targets for preventive and treatment strategies in CeVD (Pantoni, 2010). To the best of our knowledge, this is the first study to employ OCTA to assess the patterns of retinal structural and microvascular changes in patients with CeVD (Rim et al., 2020). In our study cohort, the patterns and severity of retinal structural and microvascular losses differed significantly in patients with CeVD compared with healthy controls. The patient group showed pRNFL thinning that was more severe in the temporal and nasal quadrants, and both VD and PD were significantly decreased in all quadrants in comparison with healthy controls. As expected, there was a significant correlation between visual acuity, retinal structural and microvascular parameters and transcranial doppler parameters in the CeVD group. Although our study should be interpreted as a proof-of-concept requiring independent validation in other patient groups, our data indicate the discrimination capability of OCTA with the identification of specific patterns of loss in the retinal structure and microvasculature of CeVD.

We are not aware of any study that has investigated retinal vascularization by measuring vascular density using OCTA in patients with CeVD. Wong and colleagues found that any retinal abnormalities, including arteriovenous nicking, was associated with incident stroke based on the retinal vessel caliber from fundus photographs (Wong et al., 2001). Consistent with microvascular pathology, these retinal abnormalities are likely associated with various markers of inflammation (such as white blood cell count and fibrinogen concentration) and endothelial dysfunction (such as the concentrations of von Willebrand factor and factor VIII; Wong et al., 2001). Furthermore, a wider retinal venular caliber predicted an increased risk of incident stroke, independent of traditional stroke risk factors, in a meta-analysis of 20,798 participants without diabetes (McGeechan et al., 2009). In addition, a reduction in retinal vascular fractal dimension and increased vascular tortuosity, quantified by several global geometrical parameters, have been associated with an increased risk of local ischemia (Lammie, 2002; Tomita et al., 2005). Looking at the current body of evidence, retinal microvascular lesions, including retinal vessel narrowing and widening secondary to subtle microvascular dysfunction, could be manifestations of persistent microvascular damage affecting the general vasculature, with important implications for cerebral circulation and CeVD risks.

In our study, the decreased retinal vascular density in patients with CeVD could, therefore, be explained by the impairment of the general vasculature. However, our study should carefully interpreted within patients who only suffers from CeVD. The efficacy of OCTA examinations still needs to be proved in a clinical setting, as a number of elderly people are susceptible to co-morbidities as well as the associated neuroinflammation which may easily lead to more extensive and severe retinal changes (Buga et al., 2013; Sandu et al., 2015). Further research with repeated measurements over a longer follow-up period is needed to provide more detailed information about the changes in OCTA metrics in CeVD patients with co-morbidities such as obesity, hypertension, diabetes, and age-associated retinal pathologies. In addition, larger case series are needed to investigate whether OCTA parameters bring additional predictive value to existing CeVD disease risk scores. Furthermore, the present data of visual acuity should be further improved, as logMAR (log of the Minimum Angle of Resolution) charts have been widely recognized for providing much more reliable and discriminative visual acuity measurements than Snellen charts. If our findings can be confirmed, individuals who are found to have lower retinal VD with OCTA might benefit from more stringent monitoring and therapeutic protection to reduce future vascular-related morbidity and mortality.

In summary, we showed quantitative retinal structure and microvasculature parameters could prove useful disease biomarkers for CeVD. Future work will confirm whether CeVD is indeed associated with specific patterns of loss and how the observed abnormalities in the structure and microvasculature of the retina relate to an individual’s overall microvascular status, in particular the risk of a debilitating cerebrovascular event.

## Data Availability Statement

The data analyzed in this study is subject to the following licenses/restrictions: Available from HL (e-mail, linht5@mail.sysu.edu.cn). Requests to access these datasets should be directed to HL (e-mail, linht5@mail.sysu.edu.cn).

## Ethics Statement

This study was approved by the Ethics Committee of Zhongshan Ophthalmic Center, Sun Yat-sen University (ethics board approval number: 2019KYPJ163). The patients/participants provided their written informed consent to participate in this study.

## Author Contributions

XZ, HX, and CL: equal contribution to the study. HL: conception and design. XZ, LZ, and HX: analysis and interpretation. CL, SL, HX, and XZ: data collection. PY-W-M, YZ, CC, RW, JW, TW, XW, DL, and WQ: critical revision of the manuscript. HL, HX, and PY-W-M: funding. HL and ZL: overall responsibility. All authors contributed to the article and approved the submitted version.

## Conflict of Interest

The authors declare that the research was conducted in the absence of any commercial or financial relationships that could be construed as a potential conflict of interest.

## Abbreviations

AUC: area under the curve
CCA: common carotid artery
CeVD: cerebrovascular disease
FAZ: foveal avascular zone
GC-IPL: ganglion cell-inner plexiform layer
GEEs: generalized estimating equations
ICA: internal carotid artery
OA: ophthalmic artery
OCT: optical coherence tomography
OCTA: optical coherence tomography angiography
PD: perfusion density
pRNFL: peripapillary retinal nerve fiber layer
SCP: superficial capillary plexus
VD: vessel density.

## References

[B1] Arnould L. Guenancia C. Azemar A. Alan G. Pitois S. Bichat F. (2018). The EYE-MI pilot study: a prospective acute coronary syndrome cohort evaluated with retinal optical coherence tomography angiography. *Invest. Ophthalmol. Vis. Sci.* 59 4299–4306. 10.1167/iovs.18-24090 30372758

[B2] Buga A. Di Napoli M. Popa-Wagner A. (2013). Preclinical models of stroke in aged animals with or without comorbidities: role of neuroinflammation. *Biogerontology* 14 651–662. 10.1007/s10522-013-9465-0 24057280

[B3] Cheung N. Mosley T. Islam A. Kawasaki R. Sharrett A. R. Kelin R. (2010). Retinal microvascular abnormalities and subclinical magnetic resonance imaging brain infarct: a prospective study. *Brain* 133 1987–1993. 10.1093/brain/awq127 20519327 PMC2912690

[B4] Couper D. J. Klein R. Hubbard L. D. Wong T. Y. Sorlie P. D. Cooper L. S. (2002). Reliability of retinal photography in the assessment of retinal microvascular characteristics: the atherosclerosis risk in communities study. *Am. J. Ophthalmol.* 133 78–88. 10.1016/s0002-9394(01)01315-0 11755842

[B5] DeLong E. R. DeLong D. M. Clarke-Pearson D. L. (1988). Comparing the areas under two or more correlated receiver operating characteristic curves: a nonparametric approach. *Biometrics* 44 837–845. 10.2307/2531595 3203132

[B6] Goebel W. Franke R. (2006). Retinal thickness in diabetic retinopathy: comparison of optical coherence tomography, the retinal thickness analyzer, and fundus photography. *Retina* 26 49–57. 10.1097/00006982-200601000-00009 16395139

[B7] Goto I. Katsuki S. Ikui H. Kimoto K. Mimatsu T. (1975). Pathological studies on the intracerebral and retinal arteries in cerebrovascular and noncerebrovascular diseases. *Stroke* 6 263–269. 10.1161/01.str.6.3.263 50653

[B8] Hanff T. C. Sharrett A. R. Mosley T. H. Shibata D. Knopman D. S. Kelin R. (2014). Retinal microvascular abnormalities predict progression of brain microvascular disease: an atherosclerosis risk in communities magnetic resonance imaging study. *Stroke* 45 1012–1017. 10.1161/strokeaha.113.004166 24549866 PMC4191897

[B9] Jia Y. Bailey S. T. Hwang T. S. McClintic S. M. Gao S. S. Pennesi M. E. (2015). Quantitative optical coherence tomography angiography of vascular abnormalities in the living human eye. *Proc. Natl. Acad. Sci. U.S.A.* 112 E2395–E2402. 25897021 10.1073/pnas.1500185112 PMC4426471

[B10] Kashani A. H. Chen C. L. Gahm J. K. Zheng F. Richter G. M. Rosenfeld P. J. (2017). Optical coherence tomography angiography: a comprehensive review of current methods and clinical applications. *Prog. Retin. Eye Res.* 60 66–100. 10.1016/j.preteyeres.2017.07.002 28760677 PMC5600872

[B11] Kochanek K. D. Murphy S. L. Xu J. Arias E. (2019). Deaths: final data for 2017. *Natl. Vital Stat. Rep.* 68 1–77. 32501199

[B12] Lammie G. A. (2002). Hypertensive cerebral small vessel disease and stroke. *Brain Pathol.* 12 358–370. 10.1111/j.1750-3639.2002.tb00450.x 12146804 PMC8096033

[B13] Li Y. Y. Guo R. J. Xie Y. M. Lin Y. M. Cai Y. F. Guo T. (2020). Expert consensus on Injection of Breviscapine in clinical practice. *Zhongguo Zhong Yao Za Zhi* 45 2296–2299. 32495583 10.19540/j.cnki.cjcmm.20200217.501

[B14] McGeechan K. Liew G. Macaskill P. Irwig L. Klein R. Klein E. B. K. (2009). Prediction of incident stroke events based on retinal vessel caliber: a systematic review and individual-participant meta-analysis. *Am. J. Epidemiol.* 170 1323–1332. 10.1093/aje/kwp306 19884126 PMC2800263

[B15] Menken M. Munsat T. L. Toole J. F. (2000). The global burden of disease study: implications for neurology. *Arch. Neurol.* 57 418–420. 10.1001/archneur.57.3.418 10714674

[B16] Miesfeld J. B. Brown N. L. (2019). Eye organogenesis: a hierarchical view of ocular development. *Curr. Top. Dev. Biol.* 132 351–393. 10.1016/bs.ctdb.2018.12.008 30797514

[B17] Mitchell P. Wang J. J. Wong T. Y. Smith W. Klein R. Leeder S. R. (2005). Retinal microvascular signs and risk of stroke and stroke mortality. *Neurology* 65 1005–1009. 10.1212/01.wnl.0000179177.15900.ca 16217050

[B18] Ouyang Y. Heussen F. M. Keane P. A. Sadda S. R. Walsh A. C. (2013). The retinal disease screening study: prospective comparison of nonmydriatic fundus photography and optical coherence tomography for detection of retinal irregularities. *Invest. Ophthalmol. Vis. Sci.* 54 1460–1468. 10.1167/iovs.12-10727 23322579 PMC3597191

[B19] Pantoni L. (2010). Cerebral small vessel disease: from pathogenesis and clinical characteristics to therapeutic challenges. *Lancet Neurol.* 9 689–701. 10.1016/s1474-4422(10)70104-6 20610345

[B20] Paradise M. B. Shepherd C. E. Wen W. Sachdev P. S. (2018). Neuroimaging and neuropathology indices of cerebrovascular disease burden: a systematic review. *Neurology* 91 310–320. 10.1212/wnl.0000000000005997 30021917

[B21] Rajanala A. P. Le H. T. Gill M. K. (2020). Central retinal artery occlusion as initial presentation of Moyamoya disease in a middle-aged woman. *Am. J. Ophthalmol. Case Rep.* 18:100705. 10.1016/j.ajoc.2020.100705 32322754 PMC7163072

[B22] Rim T. H. Teo A. W. J. Yang H. H. S. Cheung C. Y. Wong T. Y. (2020). Retinal vascular signs and cerebrovascular diseases. *J. Neuroophthalmol.* 40 44–59. 10.1097/wno.0000000000000888 31977663

[B23] Sandu R. E. Buga A. M. Uzoni A. Petcu E. B. Popa-Wagner A. (2015). Neuroinflammation and comorbidities are frequently ignored factors in CNS pathology. *Neural Regen. Res.* 10 1349–1355. 10.4103/1673-5374.165208 26604877 PMC4625482

[B24] Stenkamp D. L. (2015). Development of the vertebrate eye and retina. *Prog. Mol. Biol. Transl. Sci.* 134 397–414. 10.1016/bs.pmbts.2015.06.006 26310167 PMC5734922

[B25] Tomita Y. Kubis N. Calando Y. Tran D. A. Méric P. Seylaz J. (2005). Long-term in vivo investigation of mouse cerebral microcirculation by fluorescence confocal microscopy in the area of focal ischemia. *J. Cereb. Blood Flow Metab.* 25 858–867. 10.1038/sj.jcbfm.9600077 15758950

[B26] Wong T. Y. Klein R. Couper D. J. Cooper L. S. Shahar E. Hubbard L. D. (2001). Retinal microvascular abnormalities and incident stroke: the atherosclerosis risk in communities study. *Lancet* 358 1134–1140. 10.1016/s0140-6736(01)06253-5 11597667

[B27] Wong T. Y. Klein R. Sharrett A. R. Couper D. J. Klein B. E. Liao D.-P. (2002). Cerebral white matter lesions, retinopathy, and incident clinical stroke. *JAMA* 288 67–74. 10.1001/jama.288.1.67 12090864

[B28] Xiao H. Liu X. Lian P. Liao L. L. Zhong Y. M. (2020a). Different damage patterns of retinal nerve fiber layer and ganglion cell-inner plexiform layer between early glaucoma and non-glaucomatous optic neuropathy. *Int. J. Ophthalmol.* 13 893–901. 10.18240/ijo.2020.06.06 32566499 PMC7270268

[B29] Xiao H. Liu X. Liao L. Tan K. Ling Y. Zhong Y. (2020b). Reproducibility of foveal avascular zone and superficial macular retinal vasculature measurements in healthy eyes determined by two different scanning protocols of optical coherence tomography angiography. *Ophthalm. Res.* 63 244–251. 10.1159/000503071 31618736

[B30] Zhang X. Xiao H. Liu C. Zhao L. Wang J. (2020). Comparison of macular structural and vascular changes in neuromyelitis optica spectrum disorder and primary open angle glaucoma: a cross-sectional study. *Br. J. Ophthalmol.* 10.1136/bjophthalmol-2020-315842 [Epub ahead of print]. 32430343 PMC7907571

